# Familial discordance in Stargardt disease

**Published:** 2012-01-28

**Authors:** Tomas R. Burke, Stephen H. Tsang, Jana Zernant, R. Theodore Smith, Rando Allikmets

**Affiliations:** 1 Department of Ophthalmology, Edward S. Harkness Eye Institute, Columbia University, New York, NY; 2 Department of Pathology and Cell Biology, Edward S. Harkness Eye Institute, Columbia University, New York, NY; 3 Department of Biomedical Engineering, Columbia University, New York, NY; 4 The Oxford Deanery, Prince Charles Eye Unit, King Edward VII Hospital, Windsor, United Kingdom

## Abstract

**Purpose:**

To report genetic and phenotypic discordance across two generations of a family with autosomal recessive Stargardt disease (STGD1) and to compare pathogenicities of the G1961E and A1038V alleles of the ATP-binding cassette transporter, subfamily A, member 4 (*ABCA4*) gene.

**Methods:**

Five members of a family with STGD1 (patients 1–4, affected; patient 5, carrier) were included. Clinical assessment was performed together with fundus autofluorescence and spectral domain-optical coherence tomography. Patients were stratified based on the results of electroretinogram testing. Genotyping of the *ABCA4* gene was performed with the ABCR500 microarray.

**Results:**

STGD1 was diagnosed in the male proband and his female sibling (patients 1 and 2, respectively). Two children of patient 2 (patients 3 and 4) were also affected. Genotyping revealed the W663X stop mutation in all affected patients. Patients 3 and 4, who were compound heterozygous for the G1961E mutation, had earlier ages of onset than patients 1 and 2, who were compound heterozygous for the A1038V mutation. Patient 1 had an age of onset 28 years younger than patient 2, whose delayed onset can be explained by relative foveal sparing, while patient 4 had an age of onset 44 years younger than patient 2.

**Conclusions:**

The G1961E mutation, which has been considered “mild,” yields a more severe phenotype in this family than the A1038V mutation, which has been considered “severe.” Marked intrasibship discordance in clinical course is described, suggesting an additional role for modifying factors in *ABCA4* pleiotropism.

## Introduction

Autosomal recessive Stargardt disease (STGD1) is the most common cause of juvenile macular dystrophy. The disease prevalence has been estimated between one in 8–10,000 [1], but it is likely higher since the carrier frequency of mutant ATP-binding cassette transporter, subfamily A, member 4 (*ABCA4*) alleles has been determined to be as high as 1:20 [2,3]. Since the discovery of the *ABCA4* gene as the molecular cause for STGD1 [4], the phenotypic and genetic heterogeneity in this disease has been discussed extensively. While concordance of the disease phenotype within families is well documented, familial discordance, in the context of genetic homogeneity, is also a well recognized feature of *ABCA4* disease [5]. In this case study we report marked phenotypic discordance in a proband and three affected family members with STGD1, across two generations.

## Methods

**Figure 1:**
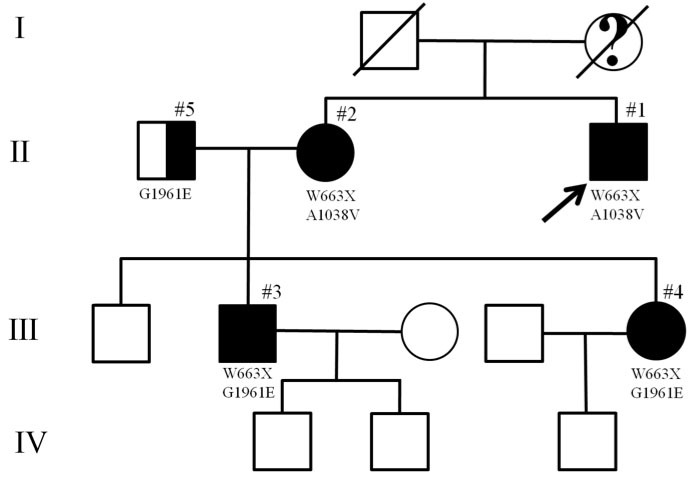
Pedigree of the family. Filled symbols indicate affected individuals, the half-filled symbol indicates a carrier for an ABCA4 mutation. The question mark in the deceased family member indicates a possible diagnosis of age-related macular degeneration.

All patients were examined at least once at the Department of Ophthalmology, Columbia University, by one of the authors (ST; Figure 1) after informed consent was obtained. None of the patients had a contributory past medical history. Age of onset was defined as the age at which visual symptoms were first reported. As three of the patients were examined on more than one occasion, the age at examination and duration of disease were recalculated for each visit. Visual acuity was measured using the Early Treatment Diabetic Retinopathy Study Chart 1. Clinical examination, fundus photography, fundus autofluorescence (FAF), and spectral domain-optical coherence tomography (SD-OCT; Heidelberg Spectralis HRA+OCT; Heidelberg Engineering, Dossenheim, Germany) were performed using standard acquisition protocols following pupil dilation with Guttae Tropicamide Minims 1% (Bausch and Lomb, Surrey, UK). The area of geographic atrophy (GA) was determined from FAF images using previously described segmentation software [6]. The percentage change in GA area from baseline was calculated for each visit. Ganzfeld full-field electroretinograms (ffERGs; Diagnosys LLC, Lowell, MA) were recorded from both eyes in accordance with the International Society for Clinical Electrophysiology of Vision standards [7]. Patients with STGD1 were grouped based on the results of their ffERGs compared with those of age similar controls [8]. The normal range was defined as the mean±2 standard deviations for age-similar controls. Those with a normal ffERG had group I disease. Genotyping was performed using the ABCR500 microarray and mutations were confirmed by direct sequencing. All research was performed with the approval of the Institutional Review Board of Columbia University.

## Results

**Figure 2:**
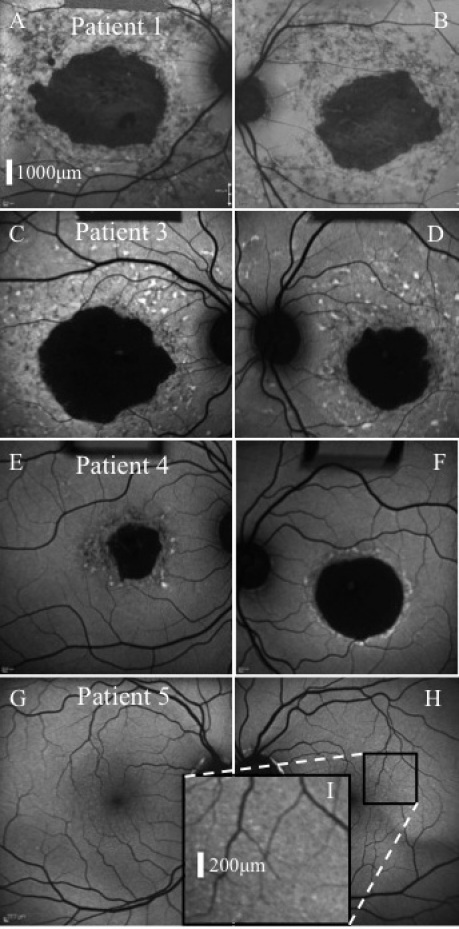
Fundus autofluorescence. Representative images of affected patients 1 (**A**, **B**), 3 (**C**, **D**), 4 (**E**, **F**), and unaffected patient 5 (**G**, **H, I**) are presented. Image **I** corresponds to the black square region in image **H**. Geographic atrophy was a prominent feature of Stargardt disease in the three affected patients, with widespread focal fundus autofluorescence (FAF) abnormalities observed in patients 1 and 3 also. Images of patient 5 revealed sparse discrete hyperautofluorescent macular lesions bilaterally. All subjects exhibited peripapillary sparing on FAF imaging. A corresponding size bar for images **A**-**H** is included in **A**. A size bar for **I** is included within the image.

**Figure 3:**
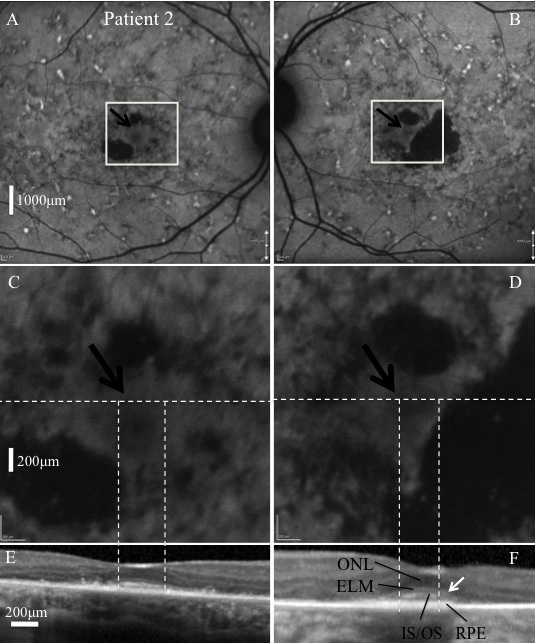
Images of patient 2. Fundus autofluorescence (FAF) images **C** and **D** correspond with the white square regions in images **A** and **B**, respectively. The horizontal white dashed lines represent the position of the spectral domain-optical coherence tomography (SD-OCT) images **E** and **F** on the corresponding en face image. FAF revealed widespread retinal disease. Relative foveal sparing was present bilaterally as evidenced by a uniform autofluorescence pattern at the foveae (black arrows). The corresponding SD-OCT images revealed relative preservation of the inner segment–outer segment junctions of the photoreceptors (IS/OS), external limiting membrane (ELM), and outer nuclear layer (ONL) in this region. The horizontal borders of the regions with preserved IS/OS are indicated by the vertical white dashed lines on the corresponding FAF and SD-OCT images. Outside these regions, absence of IS/OS was associated with functionally preserved retinal pigment epithelium (RPE), at least as identified by FAF (**C**, **D**). Qualitatively normal thickness RPE was also observed in regions with loss of IS/OS (**F**, white arrow).Corresponding size bars for **A** and **B**, **C** and **D**, and **E** and **F** are included in **A**, **C** and **E**, respectively

Demographic, clinical, and genetic information for all patients is summarized in Table 1. At examination, all five patients had normal anterior segments and intraocular pressures. Patient 2 had the highest best corrected visual acuities (BCVA) of all affected patients in the study, both at baseline and follow-up. This was explained by relative foveal sparing, present on both FAF and SD-OCT. Three of the four affected patients had widespread FAF abnormalities (i.e., focal hyperautofluorescent flecks and focal hypoautofluorescence) throughout the posterior pole, while patient 4 had abnormalities localized to the central macula (Figure 2 and Figure 3). All affected individuals had evidence of GA, and all exhibited peripapillary sparing on FAF.

**Table 1:** Summary of demographic, clinical and genetic data.

					**OD**			**OS**				
**Patient** **#, sex**	**Age at onset (years)**	**Age at exam (years)**	**Duration** **(years)**	**ERG group**	**VA**	**GA area (mm^2^)**	**% change from baseline**	**VA**	**GA area (mm^2^)**	**% change from baseline**	**Allele 1**	**Allele 2**
1, M	29	63	34	I	20/125	7.5		20/150	7		W663X	A1038V
		64	35		20/150	8.1	0.067	20/150	7.3	0.047		
		65	36		20/150	9	0.186	20/200	7.9	0.139		
2, F	57	68	11	I	20/25	0.4		20/40	1.5		W663X	A1038V
		69	12		20/30	0.5	0.309	20/30	1.7	0.167		
3, M	21	41	20	I	20/150	10.8		20/150	5.8		W663X	G1961E
4, F	13	39	26	I	20/150	2.2		20/150	6		W663X	G1961E
		40	27		20/150	2.9	0.183	20/150	7	0.109		
5, M	-	68	-	-	20/20	-		20/20	-		WT	G1961E

The W663X and A1038V mutations were detected in the *ABCA4* gene in both patients 1 and 2, the latter having an onset of symptoms at 57 years of age i.e., 28 years later than her brother. Importantly, this patient had been examined 10 years beforehand and although “subtle abnormalities in the retina” were reported, a diagnosis of STGD1 had not been made. Patients 3 and 4 inherited the W663X mutation maternally and the G1961E mutation paternally. The maximum discordance inter-sibship was 44 years. Patient 5 was entirely asymptomatic with bilateral BCVA of 20/20. Sparsely scattered hyperautofluorescent drusen-like lesions were observed throughout the posterior pole in this patient (Figure 2). No corresponding SD-OCT abnormalities were detected. All affected individuals had normal photopic and scotopic ERG amplitudes (i.e., group I disease).

## Discussion

Both patients 1 and 2 were compound heterozygous for the W663X and A1038V mutations in the *ABCA4* gene. W663X was previously reported as a disease-causing mutation [9]; it likely results in a completely dysfunctional protein due to the stop codon in the first 1/4 of the gene. A1038V, a missense mutation, is usually reported as a component of the complex allele L541P/A1038V, one of the most commonly detected mutations in the *ABCA4* gene. A1038V is also known to be pathogenic without L541P as it has a deleterious effect on ATPase by ABCA4 in vitro [10,11]. While the complex allele gives rise to an ABCA4 protein which mislocalizes within the photoreceptor with a consequent reduction in protein function, the protein associated with A1038V alone does not demonstrate mislocalization [12]. The complex allele gives rise to a broad spectrum of disease phenotypes [13-15]. The G1961E missense mutation, which has a deleterious effect on ABCA4’s ATPase activity, is the most common mutation detected in the *ABCA4* gene and has been reported to confer a milder phenotype [11,16,17].

All affected patients in this family carried the W663X mutation, and although G1961E is considered a “mild” mutation, both patients with the G1961E mutation had earlier ages of onset than those with A1038V, which is considered “severe.” A report by Cideciyan et al. describing age of disease onset in terms of “age of retina-wide disease initiation” (ADI) suggested that the G1961E mutation results in a much later ADI than the complex allele L541P/A1038V [18]. In fact, these mutations were placed on the opposite end of the ADI spectrum [18]. In this study the retinae of patients 3 and 4 (with the G1961E mutation), at ages of examination ≤41 years, demonstrated greater GA areas than the retina of patient 2 (with the A1038V mutation) at ages of examination ≥68 years. Although the percentage increase in GA area was greatest in patient 2 at follow-up, this was due to her baseline GA being the smallest of all patients.

The observed elevated pathogenicity of G1961E could be explained by a possibly “dominant negative” effect since this mutation results in inhibition, rather than stimulation, of the ATPase activity of ABCA4 by its substrate retinal [11]. The usual and expected (in a recessive disease) effect of most other missense mutations in *ABCA4* is a reduction of stimulation (a loss of function) not inhibition (a dominant negative effect) [11]. As in the study by Cideciyan et al., the number of patients in our study was too small to make definitive genotype/phenotype correlations. It can, however, be stated that G1961E does not consistently yield a milder phenotype than A1038V.

Discordance in age of onset was also seen between siblings. A 28-year difference in age of onset was documented between patients 1 and 2. This can be at least partially explained by the relative foveal sparing seen in patient 2. In a previous report of 15 families with STGD1, the maximum discordance in age of onset of symptoms was 23 years [5].

Therefore, familial discordance, in the context of genetic homogeneity, may be the result of environmental, developmental, or non-*ABCA4* genetic-modifying factors on the ABCA4 protein function [5,19]. Identification of these pathways and those that lead to foveal sparing may allow for the development of novel therapeutic strategies. Furthermore, additional research is needed for better prediction of prognosis in STGD1.
